# Antibacterial Properties of Biodegradable Silver Nanoparticle Foils Based on Various Strains of Pathogenic Bacteria Isolated from the Oral Cavity of Cats, Dogs and Horses

**DOI:** 10.3390/ma15031269

**Published:** 2022-02-08

**Authors:** Miłosz Rutkowski, Lidia Krzemińska-Fiedorowicz, Gohar Khachatryan, Julia Kabacińska, Marek Tischner, Aleksandra Suder, Klaudia Kulik, Anna Lenart-Boroń

**Affiliations:** 1 Scientific Circle of Biotechnologists “Helisa”, Microbiology Section, Department of Microbiology and Biomonitoring, Faculty of Agriculture and Economics, University of Agriculture in Krakow, 30-059 Krakow, Poland; miloszr131@gmail.com; 2 Faculty of Food Technology, University of Agriculture in Krakow, 30-149 Krakow, Poland; lidia.krzeminska@urk.edu.pl (L.K.-F.); gohar.khachatryan@urk.edu.pl (G.K.); 3 “Przychodnia Weterynaryjna Uniwersytecka” Veterinary Clinic, University Center of Veterinary Medicine, University of Agriculture in Krakow, 30-251 Krakow, Poland; julia.kabacinska@urk.edu.pl; 4 Department of Animal Reproduction, Anatomy and Genomics, Faculty of Animal Science, University of Agriculture in Krakow, 30-059 Krakow, Poland; marek.tischner@urk.edu.pl; 5 Department of Microbiology and Biomonitoring, Faculty of Agriculture and Economics, University of Agriculture in Krakow, 30-059 Krakow, Poland; aleksandra.suder@student.urk.edu.pl (A.S.); klaudia.kulik@student.urk.edu.pl (K.K.)

**Keywords:** biopolymers, animals, green chemistry, silver nanoparticles, veterinary medicine

## Abstract

Frequent occurrence of microbial resistance to biocides makes it necessary to find alternative antimicrobial substances for modern veterinary medicine. The aim of this study was to obtain biodegradable silver nanoparticle-containing (AgNPs) foils synthesized using non-toxic chemicals and evaluation of their activity against bacterial pathogens isolated from oral cavities of cats, dogs and horses. Silver nanoparticle foils were synthesized using sodium alginate, and glucose, maltose and xylose were used as reducing agents. The sizes of AgNPs differed depending on the reducing agent used (xylose < maltose < glucose). Foil without silver nanoparticles was used as control. Bacterial strains were isolated from cats, dogs and horses by swabbing their oral cavities. *Staphylococcus aureus*, methicillin-resistant *Staphylococcus aureus* (MRSA), *Escherichia coli* and extended-spectrum beta-lactamase (ESBL) producing *E. coli* were isolated on selective chromogenic microbiological media. The bactericidal effect of AgNPs foils obtained using non-toxic chemical compounds against *E. coli*, ESBL, *S. aureus* and MRSA isolated from oral cavities of selected animals was confirmed in this study. No statistically significant differences were observed between the foils obtained with different reducing agents. Therefore, all types of examined foils proved to be effective against the isolated bacteria.

## 1. Introduction

Nanotechnology is among the modern fields of science that find their wide application in human and veterinary medicine, agriculture, food and feed production, cosmetic industry, pharmacy, heritage preservation against microbial biodeterioration, textile industry and optics [1,2,3,4]. Scientific literature describes various forms of nanoparticle synthesis including physical, chemical and biological methods. However, it is the chemical method that involves the reduction of metal salts with the use of numerous reducing substances that allows for obtaining various shapes of nanoparticles [5,6,7]. Due to various shapes of the adopted structures, silver nanoparticles exhibit numerous biological effects. One of the most important properties of metal nanoparticles is their antimicrobial activity. It results from a number of actions, ultimately leading to the destruction of their biological structures and thus limiting the microbial growth. The mechanisms of antibacterial action of silver nanoparticles (AgNPs) include: disruption of cell wall and cytoplasmic membrane perforation and denaturation, denaturation of ribosomes, interruption of ATP production, cytoplasmic membrane disruption by reactive oxygen species and interference with DNA replication [8,9,10,11,12].

However, one of the widely mentioned side-effects of using nanotechnology is the possible release of nanomaterials into the environment. Furthermore, major disadvantages of popular AgNP production methods include high costs, the use of hazardous chemical materials, the demand for rigorous environmental conditions, such as pH and temperature, or the release of toxic by-products [1]. This causes understandable concerns and the need for developing a green-approach to the nanoparticle production, i.e., cost-effective, rapid, eco-friendly, scalable and not generating/using toxic substances [1,3].

Polysaccharides, many of which come from natural sources, can be described by the word “biopolymers” and are a collection of cheap and renewable raw materials used in industry to create innovative biodegradable materials [13]. The chemical structure of many polysaccharides allows for the modification of these substances using various analytical methods, including chemical and enzymatic ones. Due to the proven active properties of these biopolymers, polysaccharides of natural origin are used in the food industry and in clinical practice [14,15,16]. The possibility of using polysaccharides in the synthesis of metal nanoparticles has been documented in the literature [17,18,19]. This is because polysaccharides play an important role in the synthesis of nanometals by stabilizing the resulting particles, thus influencing their shape and size [20]. An interesting example of a biodegradable polymer is sodium alginate, which in its chemical structure is based on interconnected units of β-d-mannuronic acid and α-1-guluronic acid [21,22,23,24]. Plastic composites created with the use of sodium alginate have found application in numerous economic fields, including production of modern food packaging, in medicine, tissue engineering, in pharmacy, medicaments production and biotechnology [25,26,27].

Periodontal diseases are among the most significant problems that often affect companion animals [28]. Inflammation of the gums in animals that occurs in the oral cavity is a consequence of the immune system’s response to an emerging bacterial infection [29]. Another aspect to be considered in terms of health problems in humans and animals is the possible transmission of *Staphylococcus aureus* between humans and companion animals, due to the nasal carriage of *S. aureus* [30,31,32]. Dogs and cats, which are the most frequently kept pets, are suggested to play a role in household *S. aureus* transmission and recurrent methicillin-resistant *S. aureus* (MRSA) infections in humans [30,31]. Furthermore, Moodley et al. [32] indicate that veterinary practitioners are at significantly higher risk of MRSA carriage as a result of their professional contact with animals, e.g., horses, dogs and cats. More importantly, it has been suggested that antibiotic resistance is more frequent in canine isolates of *S. aureus* than in those of human origin [30]. *Staphylococcus aureus* is an example of a microorganism that is both a human skin and mucosa commensal but also a frequent cause of serious infections with high mortality and healthcare-associated costs [33].

Due to the fact that clinicians and veterinarians increasingly often encounter problems related to the antibiotic resistance of pathogenic microorganisms, finding alternative substances with antimicrobial activity is very essential nowadays. The phenomenon of antimicrobial resistance not only causes the problem of reducing the choice of drugs, but also contributes to the deterioration of the health and welfare of animals. The latest guidelines in veterinary medicine try to prevent this. In particular, it is worth paying attention to diseases of the oral cavity, which are one of the most common diseases that occur in companion animals. They have health consequences not only locally, but also systemically. Unfortunately, due to significant educational deficiencies in the medical and veterinary studies in the field of dentistry, there has been a lot of false information and beliefs about the treatment of oral diseases.

Therefore, the aim of this study was the synthesis of biodegradable foils containing silver nanoparticles obtained with the use of non-toxic chemicals, together with the evaluation of their antibacterial activity against pathogenic bacteria isolated from the oral cavity of companion animals.

## 2. Materials and Methods

### 2.1. Materials

Research-grade chemical reagents were used to prepare the nanocomposites, i.e., sodium alginate (Sigma-Aldrich, Poznan, Poland, molecular weight ≈ 1.565 × 10^5^ Da [34]); glycerine (Sigma-Aldrich, Poznan, Poland, 99.5%) as an excipient (plasticizer); AgNO_3_ (Sigma-Aldrich, Poznan, Poland, 99.99%); D-(+)-xylose, D-(+)-maltose monohydrate and D-(+)-glucose (Sigma-Aldrich, 99%) as reducers and deionized water. 

Microbiological media used for the experiments were as follows: Baird Parker agar, Tryptone-bile-X-glucuronide agar, Chromogenic MRSA Modified Lab Agar, Chromogenic ESBL Lab Agar and Mueller-Hinton agar, all obtained from Biomaxima (Lublin, Poland).

### 2.2. Synthesis of Alginate Films Containing Silver Nanoparticles

**Figure 1:**
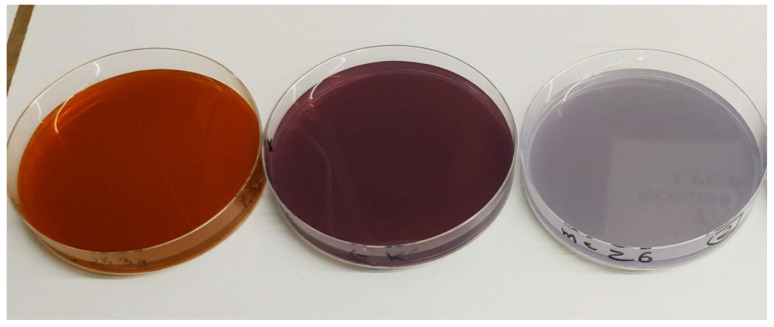
Obtained foils: from the left 1. Ag-xylose; 2. Ag-maltose; 3. Ag-glucose.

Sodium alginate (16 g) was dissolved in water so that the biopolymer concentration was 2%. The resulting suspension was gelatinized at 60 °C for 24 h. Then 200 mL of the polysaccharide gel was dispensed into four conical flasks and 2.2 mL of Tollens solution was added. Then, a glycerin solution in a ratio of 1:2 to sodium alginate was introduced into the gels as a plasticizer and heated for about 0.5 h. After this time, specific reducing agents were added to each sample. Furthermore, 8 mL of 4% xylose solution were added to sample No. 1 (Ag-xylose), 8 mL of 4% maltose solution was added to sample No. 2 (Ag-maltose) and 8 mL of 4% glucose solution was added to sample No. 3 (Ag-glucose). Sample No. 4 was designated as a control and left without the addition of reducing substance. The prepared gels were heated while stirring for an hour. After this time, individual gels were poured into dried, degreased dishes and dried in an oven for 48 h, forming nanoparticle containing foils (Figure 1).

### 2.3. Fourier Transform Infrared (FTIR) Spectroscopy

The ATR-FTIR (attenuated total reflection-Fourier transform infrared) spectra were recorded in the range of 4000–700 cm^−1^ at 4 cm^−1^ resolution using a MATTSON 3000 FT-IR spectrophotometer (Madison, WI, USA) equipped with a 30SPEC 30° reflective cap with the MIRacle ATR accessory from PIKE Technologies Inc., Madison, WI, USA.

### 2.4. Ultraviolet-Visible (UV-VIS) Spectrometry

The UV-Vis (ultraviolet-visible spectroscopy) absorption spectra were developed using a Shimadzu 2101 scanning spectrophotometer (Shimadzu, Kyoto, Japan) in the range of 200–800 nm.

### 2.5. Scanning Electron Microscopy (SEM)

The shape, size and aggregation of nanosilver was characterized using a JEOL 7550 high-resolution scanning electron microscope (SEM) (Akishima, Tokyo, Japan) equipped with a transmission electron detector (TED) (Akishima, Tokyo, Japan), retractable backscattered-electron detector (RBEI) (Akishima, Tokyo, Japan) and EDS (energy dispersive spectra) detection system of characteristic X-ray radiation INCA PentaFetx3 EDS system.

### 2.6. Isolation and Identification of Bacteria from Cats, Dogs and Horses

The presented study involves material collected from animals in the form of oral swabs. Due to the fact that the procedure involved in obtaining bacterial strains is neither harmful, nor causes any type of distress in animals, no bioethical commission approval was required for this study. 

A total of 114 randomly selected animals (46 cats, 26 dogs and 42 horses) were examined in this study by swabbing their oral cavities. After the collection of samples with sterile swabs, inoculations were performed on selective media for the isolation of bacterial pathogens and opportunistic pathogens. Baird Parker agar (Biomaxima, Lublin, Poland) was used for the isolation and identification of *Staphylococcus aureus* (grey to black colonies with clear halo after incubation for 24–48 h at 37 ± 1 °C), Tryptone-bile-X-glucuronide agar (Biomaxima, Lublin, Poland) was used for the isolation and identification of *Escherichia coli* (turquoise to blue colonies after incubation for 24 h at 44 °C), Chromogenic MRSA Modified Lab Agar (Biomaxima, Lublin, Poland) was used for the isolation of methicillin-resistant *S. aureus* (MRSA—rose to mauve colonies after incubation at 35–37 °C for 24 h) and finally Chromogenic ESBL Lab Agar (Biomaxima, Lublin, Poland) was used for the isolation of extended-spectrum beta lactamase-producing *Enterobacterales* (ESBL—*Escherichia coli*: pink to burgundy; *Klebsiella*, *Enterobacter*, *Serratia* and *Citrobacter*: green/blue to brown-green and *Proteus*, *Providensia* and *Morganella*: dark to light brown after incubation at 37 °C for 24 h). After incubation the bacterial colonies characteristic of the listed species/groups of bacteria were subcultured by plate streaking and Gram-stained preparations thereof were observed under the light microscope (1000× magnification). 

### 2.7. Evaluation of Antibacterial Activity of Nanosilver-Containing Foils

The test of the antimicrobial activity of silver nanoparticles in alginate films was carried out using a total of 79 bacterial strains, including 74 isolates collected from animals and 5 type strains (Table 1). Bacterial isolates were transferred to sterile saline solution to prepare 0.5 MacFarland suspensions, which were then streaked onto Mueller–Hinton agar (Biomaxima, Lublin, Poland). Prior to the experiment, the foils were sterilized under UV light for 20–30 min. Then, 10 × 10 mm squares were cut with a surface sterilized scissors and applied onto the surface of bacterial cultures. The cultures were incubated at 37 ± 1 °C for 24 h. Afterwards the results were read by measuring the diameters of bacterial growth inhibition zones around the foil fragments. Two diameters were read and the final result was expressed as a mean of the two reads (mm). All experiments were conducted in three replicates.

### 2.8. Statistical Analysis

The normality of the results was verified using the Shapiro–Wilk test. The distribution of the results was not close to normal, therefore non-parametric tests were applied in further analyses. The Kruskal–Wallis test was used for the following analyses:

(a) the significance of differences between the antibacterial activity of various types of foils;
(b) the significance of differences between the activity of foils against microorganisms isolated from various groups of animals;
(c) the significance of differences in the activity of foils against Gram-positive and Gram-negative bacteria;
(d) the significance of differences in the activity of foils against bacteria belonging to different species/groups.

The significance level was set at a *p* value of <0.05 for all statistical tests. All analyses were performed using Statistica ver.13.1 (2021, StatSoft, Tulsa, OK, USA).

## 3. Results and Discussion

### 3.1. Physicochemical Properties of Biodegradable Foils

**Figure 2:**
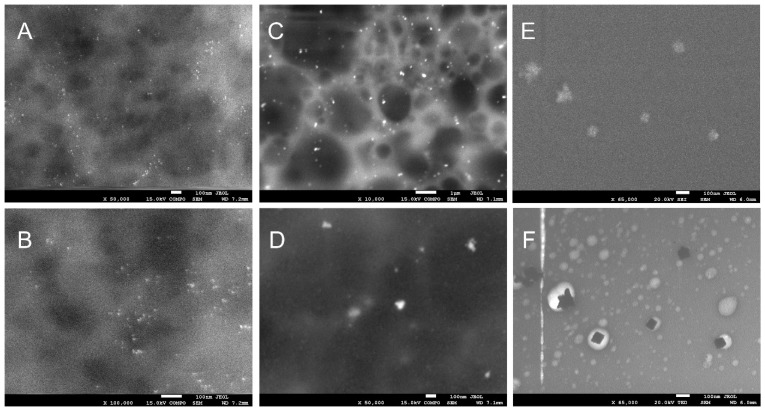
SEM micrographs of foils taken at different magnifications: (**A**,**B**) Ag-xylose (50,000 (**A**) and 100,000 (**B**) magnification); (**C**,**D**) Ag-maltose (10,000 (**B**) and 50,000 (**C**) magnification); (**E**,**F**) Ag-glucose 65,000 magnification (**E**,**F**).

In order to confirm the presence of silver nanoparticles and to determine their size, scanning electron microscopy was performed. By using secondary electron detection (in COMPO system), the presence of nanosilver in the whole structure of the obtained composites was proved (Figure 2). The resulting silver nanostructures were characterized by different sizes and shapes which depended on the used reducer.

Silver nanoparticles obtained using xylose as a reducing agent were characterized by regular and spherical shapes, their sizes varied between 5 and 10 nm. When maltose was used as a reducer, we observed an increase of the size of the nanoparticles (varying between 50 and 100 nm) and change in the shape of nanocrystals. Nanoparticles obtained using glucose formed aggregates sized approximately 100 nm on different geometrical shapes.

Morphology and stability of the silver nanoparticles depend on the method of their preparation [35], applied reducer and stabilizing reagent. Microscopic studies showed that depending on the reducer used, the obtained nanoparticles had different sizes and shapes. The shape of the nanoparticles has a strong influence on the optical and biological properties of the samples [36,37]. The used saccharides have different structures, which may explain the differences in reducing and capping ability. Filippo E. and colleagues [38] explained the influence of the structure and reducing properties of the used sugars on the reduction reaction mechanism and on the differences in the size and shape of the synthesized nanoparticles. Other scientists have shown that the reaction temperature, reaction time, the concentration of silver source, reducing agent and the amount of capping agents play vital roles in size and yields of silver nanoparticles [39]. They showed that by controlling the concentration of glucose and silver ions and by selecting the appropriate reduction reaction temperature, nanosilver of various sizes can be obtained.

**Figure 3:**
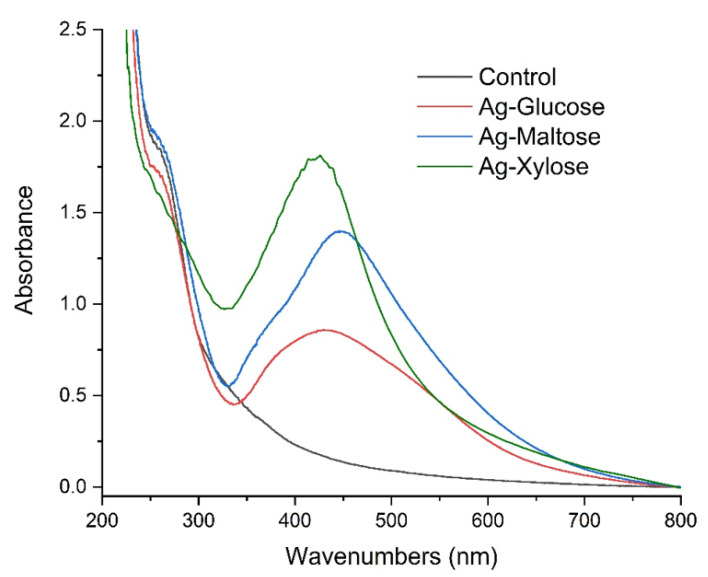
UV–Vis spectra of control (black line), Ag-xylose (green line), Ag-maltose (blue line) and Ag-glucose (red line).

Figure 3 shows the UV-Vis absorption spectra of the control sample and the Ag nanocomposites, which show an absorption band at 430 nm for Ag-xylose, 460 nm for Ag-maltose and between 375 and 520 nm for Ag-glucose. The results indicate the formation of Ag nanoparticles [34,40]. The width of the peak band indicates that the formed nanoparticles are characterized by different sizes, which has already been confirmed by the scanning electron microscope (SEM) images.

**Figure 4:**
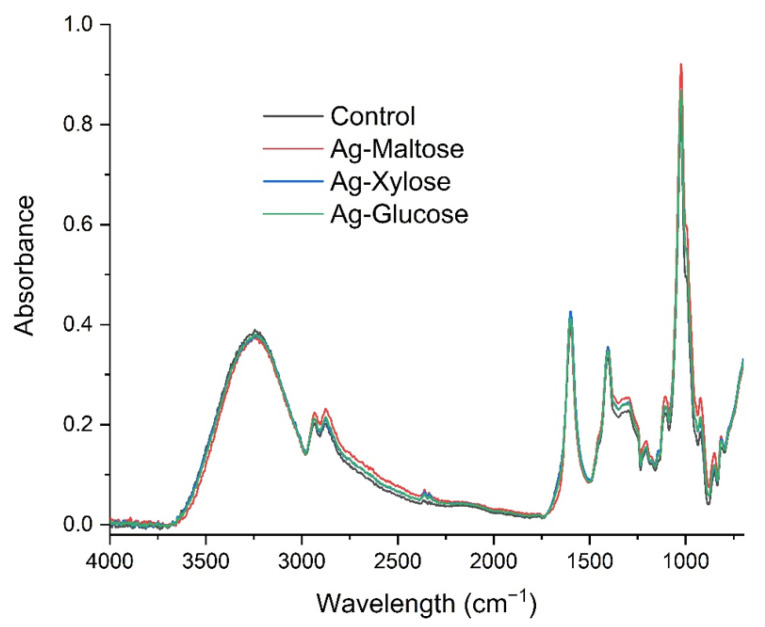
FTIR absorption spectra of control (black line), Ag-xylose (blue line), Ag-maltose (red line) and Ag-glucose (green line).

Figure 4 shows the FTIR absorption spectra of obtained bionanocomposites. We observed the characteristic spectrum of the sodium alginate with a broad band centered at approximately 3210 cm^−1^ (hydroxyl groups stretching), low intensity bands at about 2915 cm^−1^ (attributed to –CH2 groups), two peaks at 1603 cm^−1^ and 1408 cm^−1^ (asymmetric and symmetric stretching modes, respectively, of carboxylate salt groups (–COONa)), and a number of vibrations in the range of 1100–990 cm^−1^ (glycoside bonds in the polysaccharide (C–O–C stretching)) [41]. The absence of significant changes in the shape of the obtained spectra indicates that the synthesis of nanometals did not cause structural changes in the alginate molecule.

### 3.2. Isolation and Identification of Bacterial Pathogens and Opportunistic Pathogens from Animals

The examination of oral swabs collected from 114 animals (46 cats, 26 dogs and 42 horses) allowed for the isolation of a total of 74 strains of bacteria, including 28 isolates collected from cats, 24 from dogs and 22 from horses. The results indicate that 64.91% of examined animals were carriers of potentially pathogenic bacteria. The distribution of bacterial groups varied among the animals. In the case of cats *S. aureus* was the most common (*n* = 21, carried by 45.65% of cats), followed by four strains of MRSA (8.69%) and three ESBL (6.52%). Similarly, among the bacteria isolated from dogs, *S. aureus* was predominant (*n* = 21, carried by as many as 80.77% of dogs), followed by MRSA (*n* = 2, 7.69%) and ESBL (*n* = 1, 3.85%). Reversely, out of 22 equine bacterial isolates, *E. coli* was the most predominant (*n* = 15, carried by 35.71% of horses), followed by six strains of ESBL (14.29%) and one *S. aureus* (2.38%). The observed rate of canine (92.31%), feline (60.87%) and equine (52.38%) colonization by potential bacterial pathogens is higher than the one reported by, e.g., Boost et al. [30], i.e., 8.8% for dogs (with rate of isolations varying from 5.7 to 14% at various veterinary clinics) or by Bierowiec et al. [31] for cats (rate of *S. aureus* isolations of 19.17% for domestic cats without outdoor access and only 8.3% for feral cats). As for horses, no MRSA was identified by Burton et al. [42] and 7.9% of horses were carriers of methicillin-susceptible *S. aureus*. Gergeleit et al. [43] reports that the distribution of Enterobacteriaceae (including *E. coli*) is 17.8% in horses with healthy sinuses and 46.2% in horses with dental sinusitis. The possibility of transmitting microbial pathogens between animals and humans has been the subject of significant concern and has been widely described in literature [31,44,45]. However, a number of studies report that either dog or cat ownership is unlikely to significantly increase the risk of infection in healthy people (unlike in immunocompromised people) [30,31]. More importantly, both Boost et al. [30] and Bierowiec et al. [31] suggest that the bacterial transmission is more likely to occur from owner to pet animal rather than the other way round. Regardless of the potential pet-to-human transmission, another important factor to be considered is the fact that periodontal diseases in carnivorous animals (such as cats and dogs) are among the most common health issues diagnosed by veterinarians [28]. Inflammations of this region are most often caused by bacterial colonizations, including these caused by *Escherichia coli* and *Staphylococcus aureus* [46]. Having in mind the high colonization rate of the examined companion animals (particularly cats and dogs) by potentially pathogenic and harmful bacteria, coupled with their possible resistance to antimicrobial agents [30,31] and potential transmission to humans [32], it is very important to explore all options of introducing new (and possibly environmentally friendly) materials with antibacterial properties [47].

### 3.3. Antibacterial Effect of Nanosilver Foils

**Figure 5:**
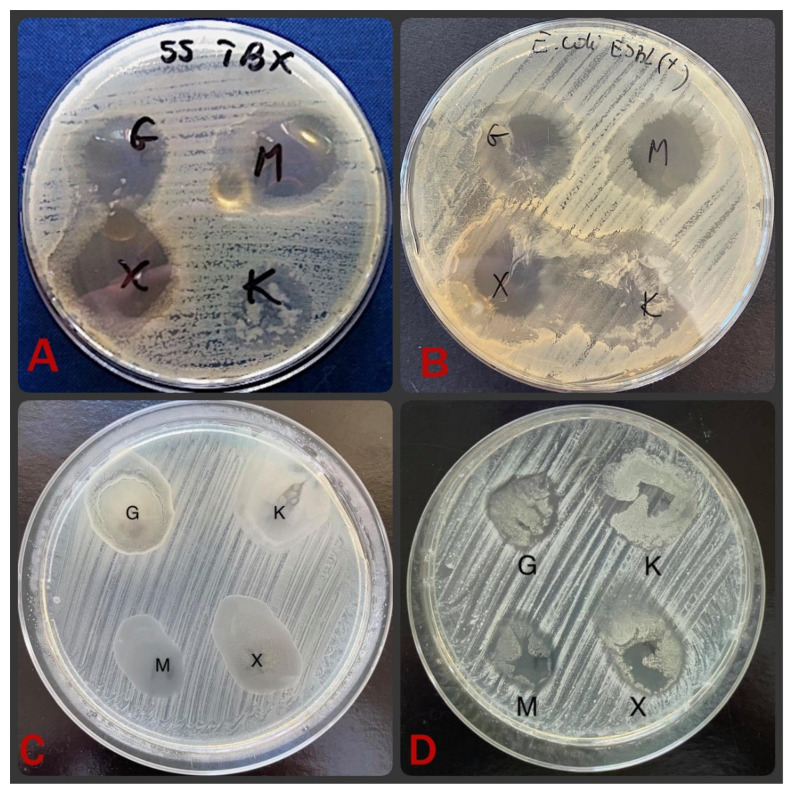
Activity of Ag-glucose (G), Ag-maltose (M), Ag-xylose (X) foils compared with control foil with sole alginate (K) against *E. coli* (**A**), ESBL-positive bacteria (**B**), *S. aureus* (**C**) and MRSA (**D**).

**Figure 6:**
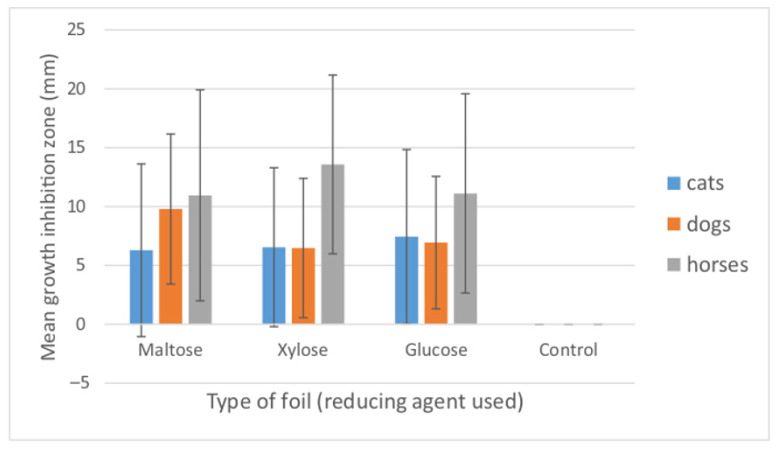
Mean growth inhibition zones (mm) caused by the three types of AgNP-containing foils produced using different reducing agents (maltose, xylose and glucose). The results are means of three replicates of tests conducted for all examined bacterial isolates (*n* = 79). Bars represent standard deviation. Control foils (sole alginate) caused no growth inhibition.

**Figure 7:**
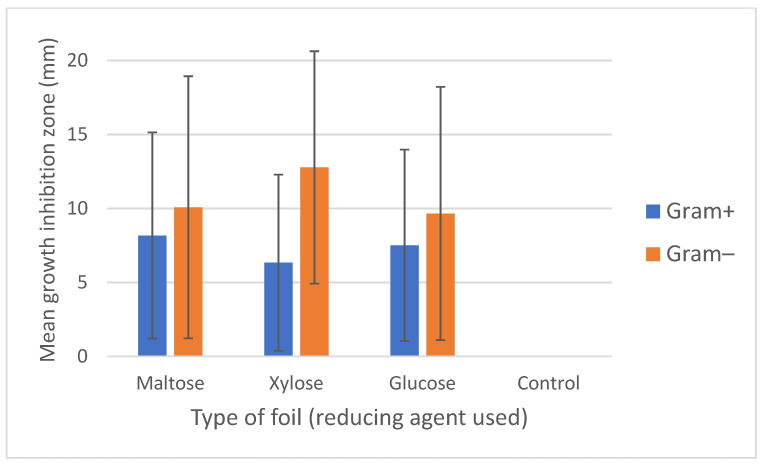
Mean growth inhibition zones (mm) caused by the three types of AgNP-containing foils produced using different reducing agents (maltose, xylose and glucose). The results are means of three replicates for the examined bacterial isolates (*n* = 79) divided into groups of Gram-positives (*n* = 51) and Gram-negatives (*n* = 28). Bars represent standard deviation. Control foils (sole alginate) caused no growth inhibition.

**Figure 8:**
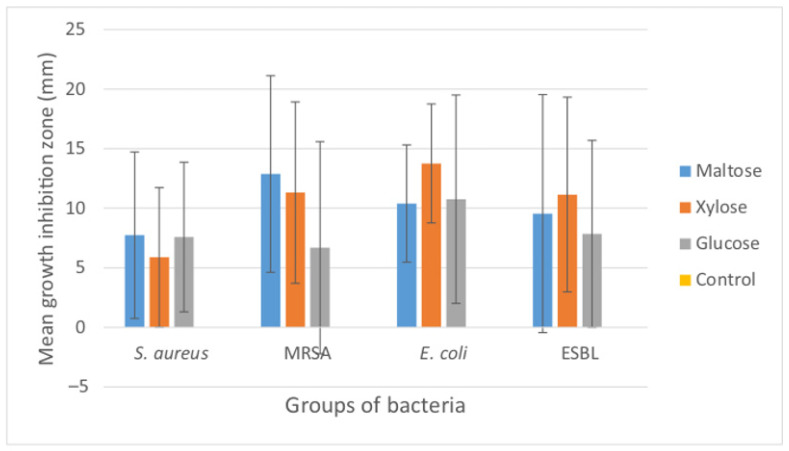
Mean growth inhibition zones (mm) caused by the three types of AgNP-containing foils produced using different reducing agents (maltose, xylose and glucose). The results are means of three replicates for the examined bacterial isolates (*n* = 79) of *S. aureus* (*n* = 45), MRSA (*n* = 6), *E. coli* (*n* = 17) and ESBL (*n* = 11). Bars represent standard deviation. Control foils (sole alginate) caused no growth inhibition.

The antibacterial effect of the AgNP foils made with three different reducing agents (maltose, glucose and xylose) and control (sole alginate) was tested against 74 strains of bacterial pathogens isolated from companion animals and 5 type species thereof (Table 1). Bacteria were divided into groups of Gram-positive (*S. aureus* and MRSA; *n* = 51) and Gram-negative (*E. coli* and ESBL; *n* = 28) strains. The results of growth inhibition zones caused by the nanosilver containing foils against each group of bacteria are presented in Figure 5, Table S1 and Table 2 and summarized in Figure 6, Figure 7 and Figure 8. Table S1 shows the mean values, standard deviations, coefficients of variation and minimum/maximum values of growth inhibition zones, for individual groups of bacteria, isolated from various animals, compared with the type strains. Table 2 shows the antibacterial efficiency of AgNP foils expressed as the percentage of bacterial isolates within various groups, whose growth was inhibited by the AgNPs. In general, the reaction of bacterial strains to the examined AgNP foils differed largely, as evidenced by the standard deviation and coefficient of variation values (Table S1). The growth inhibition zone of the *S. aureus* ATCC 29213 (susceptible to all antimicrobial agents) was higher than the mean values calculated for *S. aureus* isolates derived from cats and dogs. The type strain of MRSA (*S. aureus* NCTC 12492) showed smaller growth inhibition zones than the mean values of MRSA isolated from cats and dogs, except from the zone caused by AgNP foil produced with glucose. Both type strains of *E. coli* (ATCC 35218 and ATCC 25922) reacted very poorly to the applied AgNP foils and their growth inhibition was either none or much smaller than the mean value obtained for the strains derived from horses.

The comparison of the effect of AgNP foils against all bacteria isolated from the three groups of animals (Figure 8) shows that the growth inhibition of bacterial isolates derived from horses was the highest among all groups. As there are very few studies in which the reaction of bacterial pathogens, isolated from various animals, was examined with AgNPs further examinations are worth considering. However, it has been demonstrated that the antibiotic resistance of bacteria varies between strains isolated from various groups. For instance, Rubin et al. [48] observed significant differences in the minimum inhibitory concentrations of a number of antibiotics against *S. aureus* and *Staphylococcus pseudointermedius* of avian, bovine, equine and porcine origin. Furthermore, Middleton et al. [45] observed differences among the prevalence of methicillin resistance in *S. aureus* isolated from dogs, horses, cats and dogs. What is more, Bierowiec et al. [31] presented the differences among the prevalence of antibiotic resistant *S. aureus* between domestic and stray cats. This suggests that there can be a number of factors affecting the reaction and susceptibility of bacteria to antimicrobial agents. Among them, previous contact with antibiotics, horizontal gene transfer and colonization by already resistant bacterial strains or strains containing genetic determinants of resistance are the most important ones [31]. Moreover, there are differences in the predominant groups of bacteria isolated from various animals. Gram-negative *E. coli* and ESBL-positive bacteria dominated among horses, while Gram-positive *S. aureus* and MRSA dominated among cats and dogs. The differences in the cell wall thickness between these two groups of bacteria largely affect their reaction to antimicrobial agents [49], as discussed further in more detail. Another important aspect to refer to is that there are concerns that similarly as in the case of antibiotics, the widespread and uncontrolled use of silver nanoparticles may cause the resistance to this compound, where silver-resistant bacteria can be as problematic as antibiotic-resistant ones [50]. In this study, the efficiency of AgNP foils varied between the types of bacteria and the animal they were isolated from (Table 3). The mean antibacterial efficiency ranged between 50 and 100% (64.70–76.47% for *S. aureus*; 50–100% for MRSA; 73.33–86.67% for *E. coli* and 55–70% for ESBL).

In terms of the efficacy of action of the applied foils, no strict pattern can be observed, as the largest mean growth inhibition in strains isolated from cats was caused by the foils with glucose, in strains isolated from dogs—by the foils with maltose, whereas in strains from horses—by the foils with xylose (Figure 6).

Figure 7 shows the differences of reaction of Gram-positive (MRSA and MSSA, i.e., *S. aureus*) and Gram-negative (non-ESBL producing *E. coli* and ESBL+) bacteria to the tested foils. In all cases, Gram-negative bacteria were more susceptible to the action of AgNP foils (larger mean growth inhibition zones) than the Gram-positives. However, only differences observed for the xylose-based foils were statistically significant (*H* = 22.91; *p* = 0.000). Again, it is not possible to designate the most effective foil, as maltose-containing AgNP foil was the most effective against Gram-positive bacteria, while foil containing xylose was the most effective against Gram-negatives. Even more interestingly, the mean value of growth inhibition zone caused by the foil with xylose was the highest in the case of Gram-negative bacteria and the lowest for Gram-positives.

Further dividing the bacterial groups into *S. aureus*, MRSA, *E. coli* and ESBL showed similar results to the ones obtained for Gram-positive and Gram-negative strains grouped together (Figure 7). Both *S. aureus* and MRSA reacted most strongly to the AgNP foils with maltose, while both *E. coli* and ESBL strains reacted most strongly to the AgNP foils with xylose. Maltose-containing foil was the least effective against *E. coli*, xylose-containing foil was the least effective against *S. aureus* and glucose-containing foil was the least effective against MRSA and ESBL. Only the differences in the reaction of *E. coli* and *S. aureus* to the xylose-based AgNP foil were statistically significant (*z* = 4.84; *p* = 0.000046). Quite an important aspect of concern in terms of antibacterial efficiency of AgNPs is their shape and size, which varied among the examined foils with the following pattern: Ag-xylose < Ag-maltose < Ag-glucose. According to Wei et al. [35], the AgNPs’ sizes and shapes are among the most important factors affecting their toxicity to living cells with a general conclusion that the smaller the size of AgNPs, the stronger the cytotoxic effect they could have. The pattern of size differences among the AgNPs examined in this study was directly followed by the bacterial growth inhibition pattern only in the case of ESBL-positive bacteria. The highest growth inhibition caused by Ag-xylose was also observed in *E. coli*, but then Ag-glucose caused the second most effective inhibition. It was on *E. coli* that thorough research was carried out concerning the mechanisms of AgNPs’ bactericidal actions [36], reporting that AgNPs increase the outer membrane permeability to toxic substances and cause leakage of cellular materials.

Similar differences in the reaction of Gram-negative *E. coli* and Gram-positive *S. aureus* to the effect of photoactivated AgNPs were observed by Al-Sharqi et al. [49], with much stronger antibacterial effect of AgNPs against *E. coli*. They attributed these differences to the structure of bacterial cell walls, i.e., thicker cell wall of Gram-positive bacteria and stronger negative charge of cell walls in Gram-negatives, which promotes adhesion of AgNP to the bacterial cell walls and thus higher effectiveness of silver nanoparticles against bacteria.

Notwithstanding all the above, the antibacterial effectiveness of all three types of AgNP foils is statistically significant as compared to the control (*p* < 0.05, Table 3), regardless of the reducing agent used and the type of bacteria tested. The well-known antibacterial properties of AgNPs, which result from perforation/disruption of cell membranes, generation of reactive oxygen species responsible for cell lysis and interference with vital biomolecules, ribosome function interference, DNA translation alteration and inhibition of DNA replication [8], have been confirmed in the case of the biodegradable AgNP foils, examined in this study.

## 4. Conclusions

The conducted studies confirmed that the oral cavities of animals, such as cats, dogs and horses, are inhabited by bacterial pathogens, such as methicillin-resistant *Staphylococcus aureus* (MRSA) and extended spectrum beta-lactamase producing *E. coli* (ESBL), as well as by opportunistic pathogens, such as methicillin-susceptible *S. aureus* and non-ESBL producing *E. coli*. The physicochemical analyses confirmed the successful formation of Ag nanoparticles using all types of non-toxic, biodegradable reducing agents, such as glucose, maltose and xylose. The sizes of AgNPs varied and increased as follows: Ag-xylose < Ag-maltose < Ag-glucose. In our study, the Ag-xylose particle size smaller than 10 nm proved to be the most effective against Gram-negative bacteria.

All types of silver-nanoparticle-containing foils proved to cause growth inhibition of the potential bacterial pathogens. The efficiency of growth inhibition varied between the species of bacteria. AgNP foils produced using glucose as reducing agent were most effective against bacteria isolated from cats (71.43% efficiency), foils produced using maltose as reducing agent were the most effective against bacteria isolated from dogs (85.71–100% efficiency), whereas foils produced using xylose were the most effective against bacteria isolated from horses (83.3–100% efficiency). AgNP foils produced using xylose were the most effective against *E. coli* and ESBL, while foils produced using maltose were the most effective against *S. aureus* and MRSA. Only in the case of ESBL was the growth inhibition directly proportional to the changes in AgNPs’ sizes. The obtained results suggest that the examined non-toxic, biodegradable silver nanoparticle foils proved effective against the potential pathogenic bacteria isolated from cats, dogs and horses.

Therefore, future studies in terms of the application of the examined AgNP foils should be first focused on their non-toxicity to animal mucosa and gums, followed by their applicability as modern dressings, gels, toothpaste or rinsing solution in veterinary medicine.

## Figures and Tables

**Table 1:** Characteristics of bacterial strains used in the experiment.

Bacterial Species/Groups	Cats	Dogs	Horses	Type Species
*S. aureus*	21	21	1	*S. aureus* ATCC 29213 (susceptible)
MRSA	4	2	0	*S. aureus* NCTC 12493
*E. coli*	0	0	15	*E. coli* ATCC 35218*E. coli* ATCC 25922
ESBL	3	1	6	*E. coli* ESBL (+) UTI
Total	28	24	22	5

**Table 2:** Percentage (%) of bacterial isolates, whose growth was inhibited by the AgNPs.

Animals	Bacteria	AgNP Foils		
		Maltose	Xylose	Glucose
cats	*S. aureus*	57.14	66.67	71.43
	MRSA	100.00	100.00	100.00
	ESBL	16.67	33.33	33.33
dogs	*S. aureus*	85.71	61.90	80.95
	MRSA	100.00	100.00	0.00
	ESBL	100.00	100.00	0.00
horses	*S. aureus*	0.00	100.00	100.00
	*E. coli*	73.33	86.67	73.33
	ESBL	75.00	83.33	75.00
total	*S. aureus*	70.59	64.71	76.47
	MRSA	100.00	100.00	50.00
	*E. coli*	60.00	70.00	55.00
	ESBL	70.59	64.71	76.47

**Table 3:** Results of Kruskal–Wallis test of significance of differences in antibacterial effects of AgNP foils obtained with various reducing agents.

Groups of Bacteria	Tested Foils				
	Type of Foil	Ag-Maltose	Ag-Xylose	Ag-Glucose	Control
*S. aureus* *H* = 109.53 *p* = 0.000	Ag-maltose	-	0.89/1.00	0.32/1.00	8.21/0.000
	Ag-xylose	0.89/1.00	-	1.22/1.00	7.66/0.000
	Ag-glucose	0.32/1.00	1.22/1.00	-	8.52/0.000
	Control	8.21/0.000	7.66/0.000	8.52/0.000	-
MRSA *H* = 16.99 *p* = 0.0007	Ag-maltose	-	0.51/1.00	1.92/0.790	3.65/0.006
	Ag-xylose	0.51/1.00	-	1.41/1.00	3.14/0.007
	Ag-glucose	1.92/1.00	1.41/1.00	-	1.73/1.00
	Control	3.65/0.009	3.14/0.007	1.73/1.00	-
*E. coli* *H* = 21.69 *p* = 0.0001	Ag-maltose	-	1.58/0.691	0.28/1.00	4.76/0.000
	Ag-xylose	1.58/0.691	-	1.30/1.00	6.34/0.000
	Ag-glucose	0.28/1.00	1.30/1.00	-	5.04/0.000
	Control	4.76/0.000	6.34/0.000	5.04/0.030	-
ESBL *H* = 50.13 *p* = 0.000	Ag-maltose	-	0.59/1.00	0.59/1.00	3.36/0.005
	Ag-xylose	0.59/1.00	-	1.18/1.00	3.95/0.000
	Ag-glucose	0.59/1.00	1.18/1.00	-	2.77/0.034
	Control	3.36/0.005	3.95/0.000	2.77/0.034	-

## Data Availability

Data sharing is not applicable to this article.

## Supplementary Materials

The following are available online at www.mdpi.com/article/10.3390/ma15031269/s1, Table S1: Growth inhibition zones in bacterial pathogens and opportunistic pathogens caused by the silver nanoparticle foils (mm). The results are means of three replicates. CV (%)—coefficient of variation; min/max—the smallest and the largest diameter of bacterial growth inhibition (mm) caused by the AgNP foils against each group of bacteria.
